# DnaJ-PKAc fusion induces liver inflammation in a zebrafish model of fibrolamellar carcinoma

**DOI:** 10.1242/dmm.042564

**Published:** 2020-04-30

**Authors:** Sofia de Oliveira, Ruth A. Houseright, Benjamin G. Korte, Anna Huttenlocher

**Affiliations:** 1 Department of Medical Microbiology and Immunology, University of Wisconsin-Madison, Madison, WI 53706, USA; 2 Department of Pediatrics, University of Wisconsin-Madison, Madison, WI 53792, USA

**Keywords:** Fibrolamellar carcinoma, Liver, Inflammation, Early progression, Non-invasive imaging

## Abstract

Fibrolamellar carcinoma (FLC) is a rare liver cancer that affects adolescents and young adults. Genomic analysis of FLC has revealed a 400 kb deletion in chromosome 19 that leads to the chimeric transcript *DNAJB1-PRKACA* (DnaJ-PKAc), comprised of the first exon of heat shock protein 40 (*DNAJB1*) and exons 2-10 of the catalytic subunit of protein kinase A *(PRKACA*). Here, we report a new zebrafish model of FLC induced by ectopic expression of zebrafish Dnaja-Pkaca (zfDnaJa-Pkaca) in hepatocytes that is amenable to live imaging of early innate immune inflammation. Expression of zfDnaJa-Pkaca in hepatocytes induces hepatomegaly and increased hepatocyte size. In addition, FLC larvae exhibit early innate immune inflammation characterized by early infiltration of neutrophils and macrophages into the liver microenvironment. Increased Caspase-a (the zebrafish homolog for human caspase-1) activity was also found in the liver of FLC larvae, and pharmacological inhibition of Tnfα and caspase-a decreased liver size and inflammation. Overall, these findings show that innate immune inflammation is an early feature in a zebrafish model of FLC and that pharmacological inhibition of TNFα or caspase-1 activity might be targets to treat inflammation and progression in FLC patients.

This article has an associated First Person interview with the first author of the paper.

## Abstract (precis)

**Summary:** Inflammation and Caspase-a activation occur early in a new zebrafish model for fibrolamellar carcinoma (FLC). Pharmacological inhibition of TNFα secretion and caspase-1 might be useful approaches to treat FLC patients.

## INTRODUCTION

Fibrolamellar carcinoma (FLC) is a rare and understudied liver cancer that primarily affects adolescents and young adults. Surgery (resection/liver transplantation) is the most common treatment for FLC patients. Recurrence is very common in FLC patients and, unfortunately, the therapeutic options are not very effective. Therefore, studies have focused on identifying the molecular mechanisms that drive the disease (Dhingra et al., 2010; Li et al., 2010; Ross et al., 2011; Simon et al., 2015; Honeyman et al., 2014; Oikawa et al., 2015; Graham et al., 2015; Dinh et al., 2019, 2017; Riehle et al., 2019; Riggle et al., 2016; Turnham et al., 2019). There is only one unique molecular target responsible for driving the disease, the *DNAJB1-PRKACA* (referred to as DnaJ-PKAc) fusion transcript that results from a 400 kb deletion on chromosome 19 (Cornella et al., 2015; Dinh et al., 2017; Honeyman et al., 2014; Simon et al., 2015). Indeed, recently developed murine models using CRISPR/Cas9 or overexpression of this fusion transcript have shown that DnaJ-PKAc is sufficient to drive tumorigenesis *in vivo* (Engelholm et al., 2017; Kastenhuber et al., 2017).

The chimeric enzyme is comprised of the J-domain of the chaperonin-binding domain of heat shock protein 40 or DnaJ (the amino-terminal 69 residues) fused to the carboxyl-terminal 336 residues of PKAc (Riggle et al., 2016; Simon et al., 2015; Xu et al., 2015; Honeyman et al., 2014). Importantly, this chimeric enzyme retains its enzymatic activity and is overexpressed in FLC (Graham et al., 2018; Riggle et al., 2016; Honeyman et al., 2014). In addition, this fusion protein can also function as a scaffold that recruits heat shock protein 70, a component that is frequently upregulated in cancers (Murphy, 2013). This scaffold further recruits the RAF/MEK/ERK signaling complex and promotes increased ERK signaling and proliferative growth (Turnham et al., 2019). The changes in PKA signaling have been implicated in driving specific gene expression signatures including altered non-coding RNAs (Farber et al., 2018; Dinh et al., 2017). MicroRNAs are also dysregulated in FLC, with downregulation of the known tumor suppressor miR-375 (Dinh et al., 2019), providing a potential therapeutic target.

PKA is known to regulate both the innate and adaptive immune responses (Serezani et al., 2008; Skalhegg et al., 2005). However, how aberrant PKA signaling in FLC affects innate immunity remains unclear. This is particularly important because of a growing interest in understanding the immune response to cancer and increasing evidence showing that inflammation plays a key role in liver cancer development and progression (de Oliveira et al., 2019; Kuang et al., 2009; Kuang et al., 2011; Li et al., 2015; Yan et al., 2015, 2017). Here, we utilized zebrafish to model FLC and to image the effects of DnaJ-PKAc on liver morphology and inflammation. Zebrafish have unmatched live-imaging capabilities and scalability, and they are amenable to whole-organism-level experiments and genetic and pharmacological perturbations. Hepatocyte-specific overexpression of the *dnajb1a-prkcaa* fusion transcript promotes hepatocellular atypia suggestive of malignancy in larvae and the formation of masses in some adults. In addition, expression of DnaJ-PKAc induces infiltration of neutrophils and macrophages into the liver area in transgenic larvae. Finally, pharmacological inhibition of Tnfα or Caspase-a decreased neutrophil and macrophage infiltration and liver size in FLC larvae. Overall, our data suggest that inflammation occurs early in FLC larvae and that pharmacological inhibition of TNFα secretion and caspase-1 activity might be targets to treat inflammation and progression in FLC patients.

## RESULTS

### Overexpression of Dnaj-Pkaca in hepatocytes can induce mass formation in the liver of adult zebrafish

**Fig. 1.**
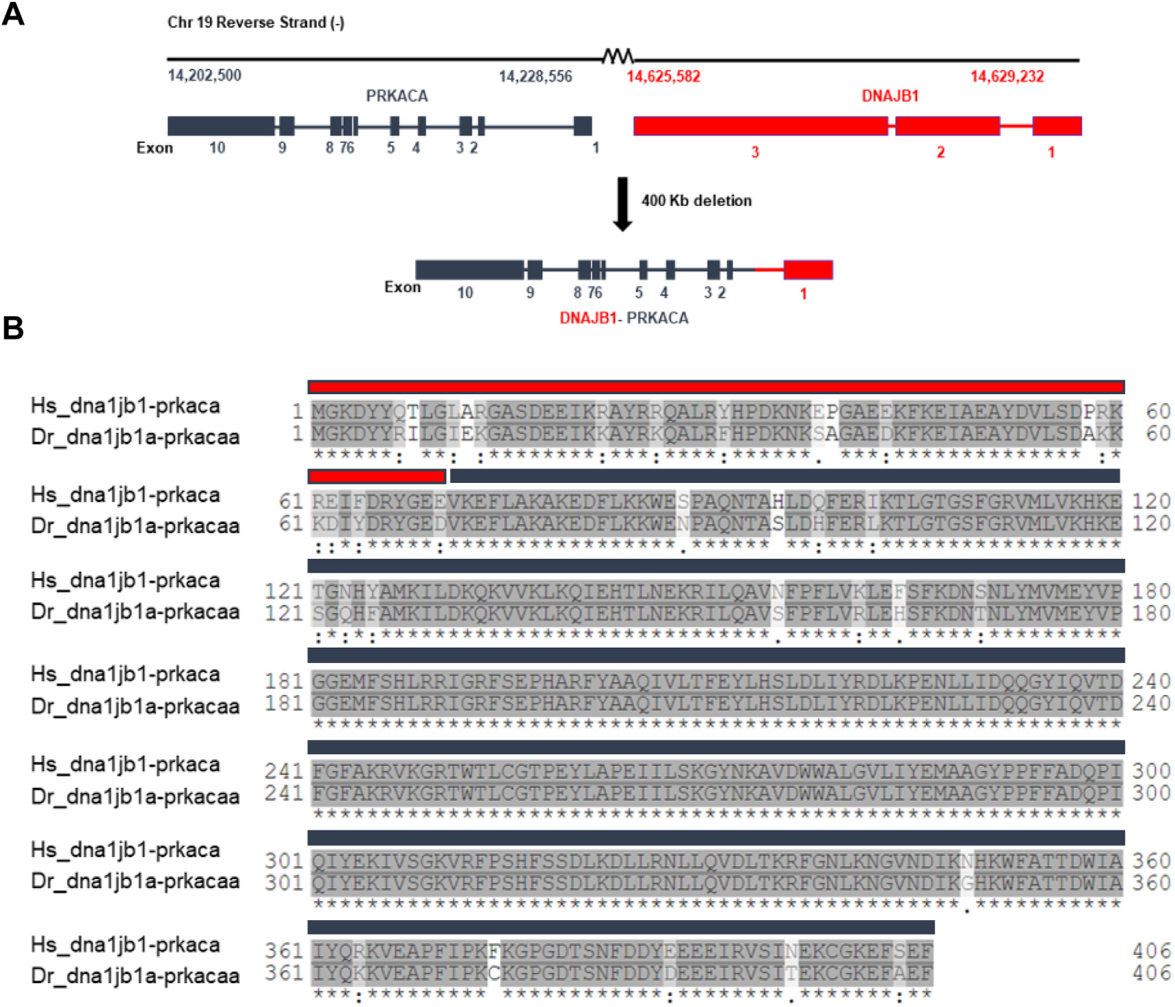
**Schematic of *DNAJB1-PRKACA* (DnaJ-PKAc) chimera in fibrolamellar carcinoma (FLC).** (A) Chromosome position, exon/intron diagram of *DNAJB1* and *PRKACA* genes, and fusion product after deletion of 400 kb observed in FLC patients. (B) Clustal Omega alignment of human (Hs) and zebrafish (Dr) amino acid sequences corresponding to exon 1 of *dnajb1a* (red) and to exons 2-10 of *prkacaa* (black) (identity, 91.6%; similarity, 97%).

**Fig. 2.**
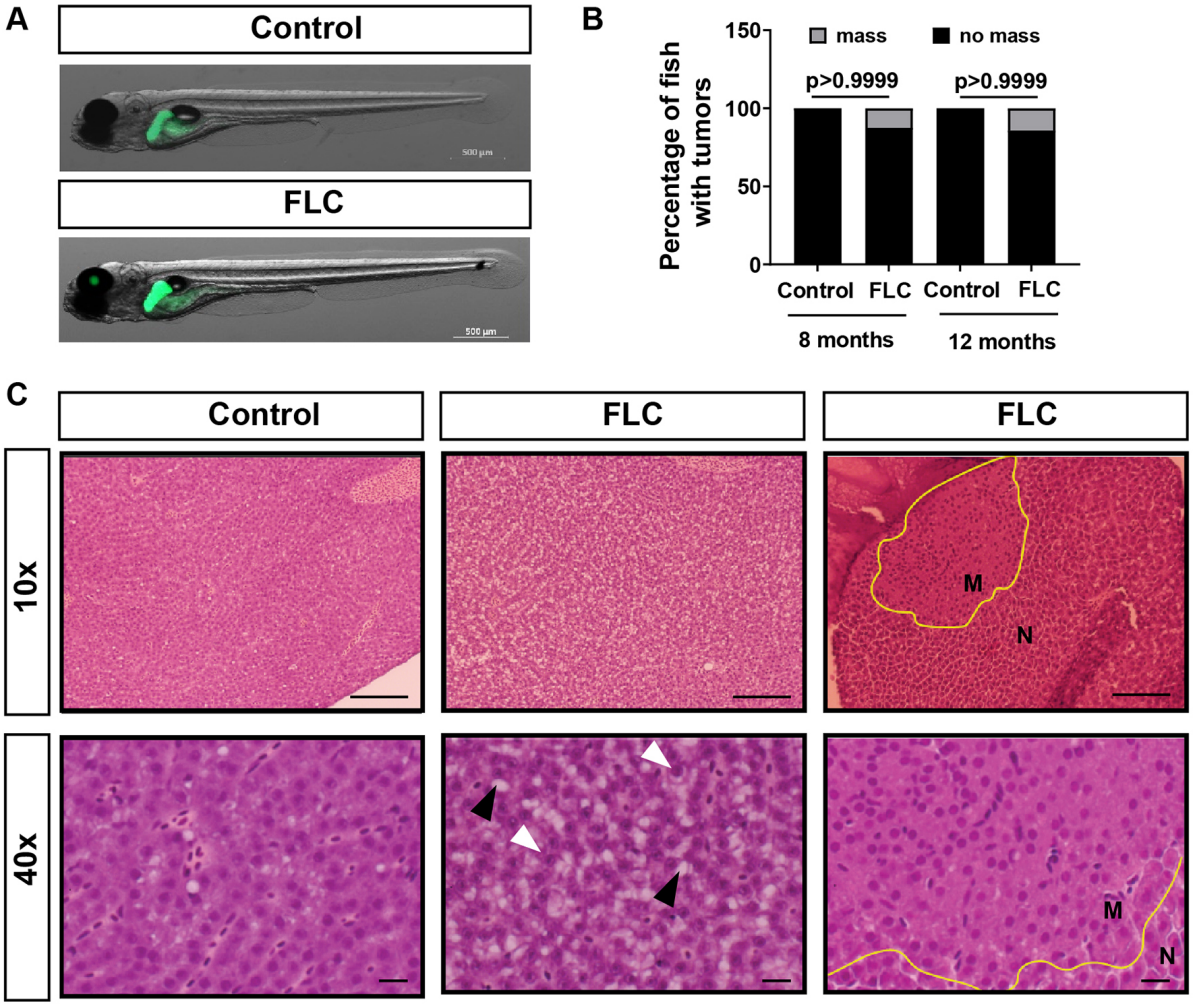
**Overexpression of Dnajb1a-Prkacaa** **(zfDnaJa-Pkaca)** **can induce liver masses at adult stages.** (A) Representative maximum-intensity projections (MIPs) of 7 day post-fertilization (dpf) FLC larvae [*Tg(fabp10a:dnajb1a-prkacaa)/Tg(fabp10a:egfp-l10a)*] and control siblings [*Tg(fabp10a:egfp-l10a)*]. (B) Chi-square graphs showing percentages of fish with masses (8 months, control *N*=5, FLC *N*=8; 12 months, control *N*=5, FLC *N*=7). (C) Representative images of H&E staining of the livers of 12-month-old fish at 10× and 40× magnification. Yellow outlines delineate masses, white arrowheads indicate prominent nucleoli and black arrowheads indicate lipid vacuoles. Scale bars: 60 μm (10× magnification) and 10 μm (40× magnification). M, mass; N, normal tissue.

The *DNAJB1-PRKACA* chimera in FLC (Fig. 1A) is sufficient to drive tumorigenesis *in vivo* in murine models (Engelholm et al., 2017; Kastenhuber et al., 2017). To determine whether the fusion transcript drives mass formation in zebrafish, we generated a fusion of zebrafish *dnajb1* and *prkaca*. Owing to whole-genome duplication (Meyer and Schartl, 1999), zebrafish have two homologous genes for *dnajb1*, *dnajb1a* (ENSDARG00000099383) and *dnajb1b* (ENSDARG00000041394), located on chromosomes 3 and 1, respectively. In addition, there are also two homologous genes for *prkaca*, *prkacaa* (ENSDARG00000100349) and *prkacab* (ENSDARG00000016809), also located on chromosomes 3 and 1, respectively. Using the hepatocyte-specific *fabp10a* promoter, we overexpressed the zebrafish *dnajb1a-prkacaa* fusion transcript (referred to as zfDnaJa-Pkaca), with 91.6% identity and 97% similarity with its human counterpart (Fig. 1B). Using the transposase system, we generated a stable line, *Tg(fabp10a:dnajb1a-prkacaa_cryaa:Cerullean)*, in the pigment-deficient Casper background to enable non-invasive live imaging at later developmental stages*.* To facilitate liver visualization, we outcrossed the FLC line to a transgenic line expressing *egfp-l10a*, *Tg(fabp10a:egfp-l10a)* (Table 1, Fig. 2A). To determine if ectopic expression of *dnajb1a-prkacaa* fusion transcript was sufficient to induce tumorigenesis in zebrafish, we dissected livers from 8- and 12-month-old FLC and control fish and performed a blinded, conventional histopathological evaluation of Hematoxylin and Eosin (H&E)-stained sections. Compared with controls, FLC livers were larger and displayed mildly disrupted hepatocellular architecture, characterized by increased thickness of hepatocellular cords. Hepatocytes from FLC livers also had vesiculated chromatin and prominent and sometimes multiple nucleoli (Fig. 2C). In 1/8 fish at 8 months and an additional 1/7 fish at 12 months (Fig. 2B), unencapsulated masses were noted within the hepatic parenchyma that were not noted in control fish. Masses lacked typical hepatic architecture and consisted of disorganized sheets of well-differentiated hepatocytes traversed by blood vessels (Fig. 2C). Staining with Masson's trichrome showed no difference in collagen deposition between FLC livers and controls (data not shown). Our findings suggest that zfDnaJa-Pkaca can induce hepatomegaly and liver mass formation *in vivo* in adult zebrafish.

**Table 1.**
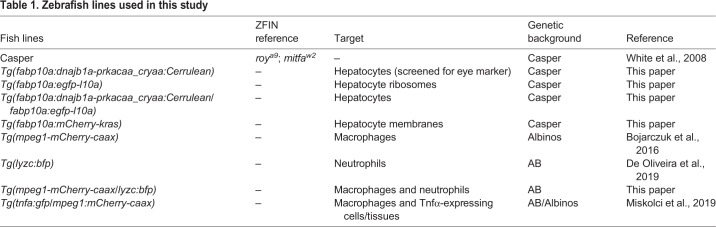
**Zebrafish lines used in this study**

### FLC larvae display early malignancy features

**Fig. 3.**
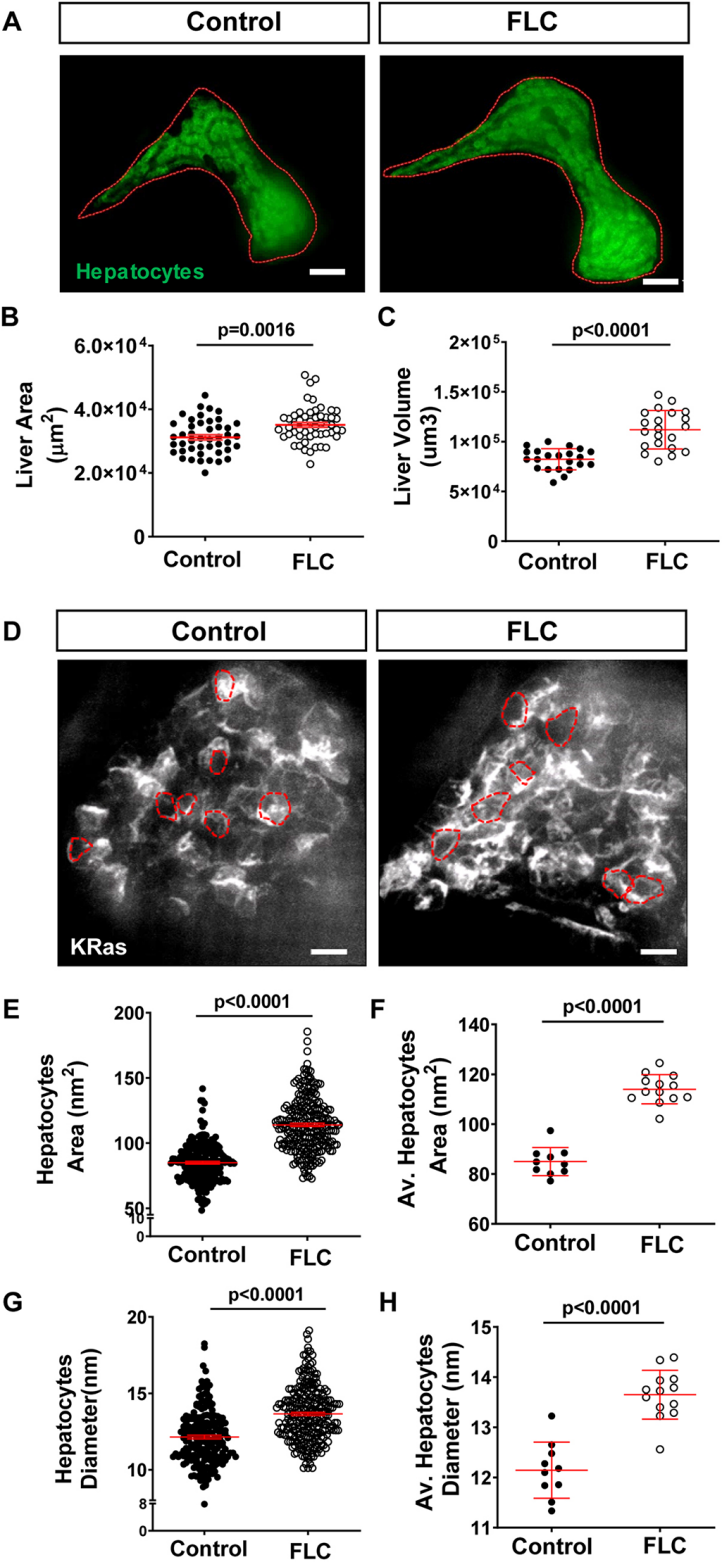
**Overexpression of** **zfDnaJa-Pkaca** **modulates liver morphology.** (A) Representative MIPs of 7 dpf FLC larvae [*Tg(fabp10a:dnajb1a-prkacaa)/Tg(fabp10a:egfp-l10a*)] and control siblings [*Tg(fabp10a:egfp-l10a)*]. Dotted red lines indicate liver area. (B,C) Graphs showing liver area (B) (control *N*=45, FLC *N*=53) and liver volume (C) (control *N*=22, FLC *N*=21). (D) Representative MIPs of 7 dpf FLC larvae [*Tg(fabp10a:dnajb1a-prkacaa)/Tg(fabp10a:mCherry-kras)*] and control siblings [*Tg(fabp10a:mCherry-kras)*]. Dashed red lines indicate hepatocyte area. (E-H) Graphs showing hepatocyte area (E,F) and diameter (G,H). In E and G, each dot represents one hepatocyte (control *N*=201, FLC *N*=261); in F and H, each dot represents one larva (control *N*=10, FLC *N*=13). Scale bars: 20 μm. Data are from at least three independent experiments. Analysis performed in EMM in R. Dot plots show mean±s.e.m.; *P*-values are shown on graphs.

Zebrafish larvae are a valuable model to study the early cellular and molecular events involved in liver cancer progression (de Oliveira et al., 2019; Huang et al., 2017; Nguyen et al., 2012; Yan et al., 2015, 2017; Zhao et al., 2016). Next, we wanted to determine if overexpression of zfDnaJa-Pkaca induced morphologic changes in the liver that suggest cell transformation and early malignancy. Liver size is a common measurement used to quantify liver disease progression (de Oliveira et al., 2019; Evason et al., 2015; Yan et al., 2015). Liver area and liver volume were increased in 7 days post-fertilization (dpf) FLC larvae compared to control siblings (Fig. 3A-C). We next took advantage of the optical accessibility of zebrafish larvae to evaluate the size of hepatocytes *in vivo* by non-invasive live imaging. We outcrossed the FLC transgenic line, *Tg(fabp10a:dnajb1a-prkacaa_cryaa:Cerullean)*, with a line that expresses Kras in the hepatocyte membrane, *Tg(fabp10a:mCherry-kras)* (Table 1)*.* In FLC larvae, we observed an increase in hepatocyte area and diameter (Fig. 3D-H). Altogether, these data suggest that ectopic expression of zfDnaJa-Pkaca in hepatocytes induces hepatomegaly, suggesting that 7 dpf FLC larvae can be used to study early FLC progression.

### FLC larvae display innate immune cell infiltration in the liver area

**Fig. 4.**
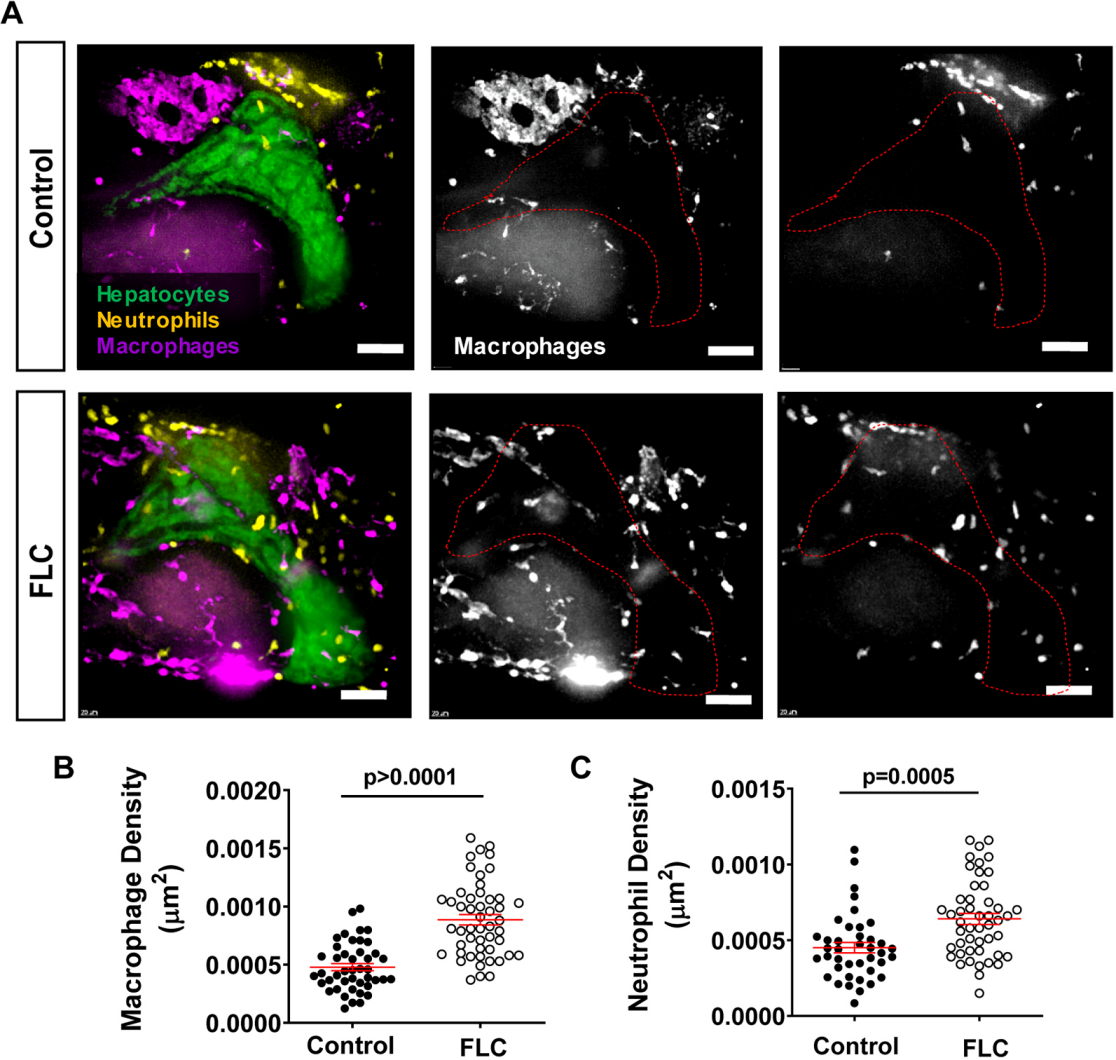
**Overexpression of** **zfDnaJa-Pkaca** **induces liver inflammation.** (A) Representative MIPs of 7 dpf FLC larvae [*Tg(fabp10a:dnajb1a-prkacaa)/Tg(fabp10a:egfp-l10a)/Tg(lyzc:bfp)/Tg(mpeg1:mCherry-caax)*] and control siblings [*Tg(fabp10a:egfp-l10a)/Tg(lyzc:bfp)/Tg(mpeg1:mCherry-caax)*]. Dotted red lines indicate liver area. (B,C) Graphs showing macrophage (B) (control *N*=44, FLC *N*=50) and neutrophil (C) (control *N*=40, FLC *N*=48) density in the liver area. Scale bars: 40 μm. Data are from at least three independent experiments. Analysis performed in EMM in R. Dot plots show mean±s.e.m.; *P*-values are shown on graphs.

PKA is a known regulator of the immune response (Serezani et al., 2008; Skalhegg et al., 2005). However, it is still unclear how DnaJ-PKAc affects the immune cell composition in the liver microenvironment. To address this question, we outcrossed the FLC transgenic fish with labeled hepatocytes, *Tg(fabp10a:dnajb1a-prkacaa_cryaa:Cerullean)/(fabp10a:egfp-l10a)*, with the double-transgenic neutrophil- and macrophage-labeled line, *Tg(mpeg1-mCherry-caax/lyzc:bfp)* (Table 1) (Fig. 4A). We found that overexpression of the aberrant PKA increased both neutrophil and macrophage infiltration into the liver area in FLC transgenic larvae compared to control larvae (Fig. 4A-C). Time-lapse movies of the liver microenvironment area (Movies 1 and 2) revealed robust recruitment of neutrophils to livers of FLC transgenic larvae compared to control larvae at this early phase. Livers of FLC larvae also exhibited an increased presence of macrophages in association with transformed hepatocytes, some of which exhibited a round shape (Movies 1 and 2). Overall, these data suggest that the presence of zfDnaJa-Pkaca triggers an inflammatory response in the liver of FLC larvae.

### FLC larvae have increased pro-inflammatory macrophages and Caspase-a activity in the liver

**Fig. 5.**
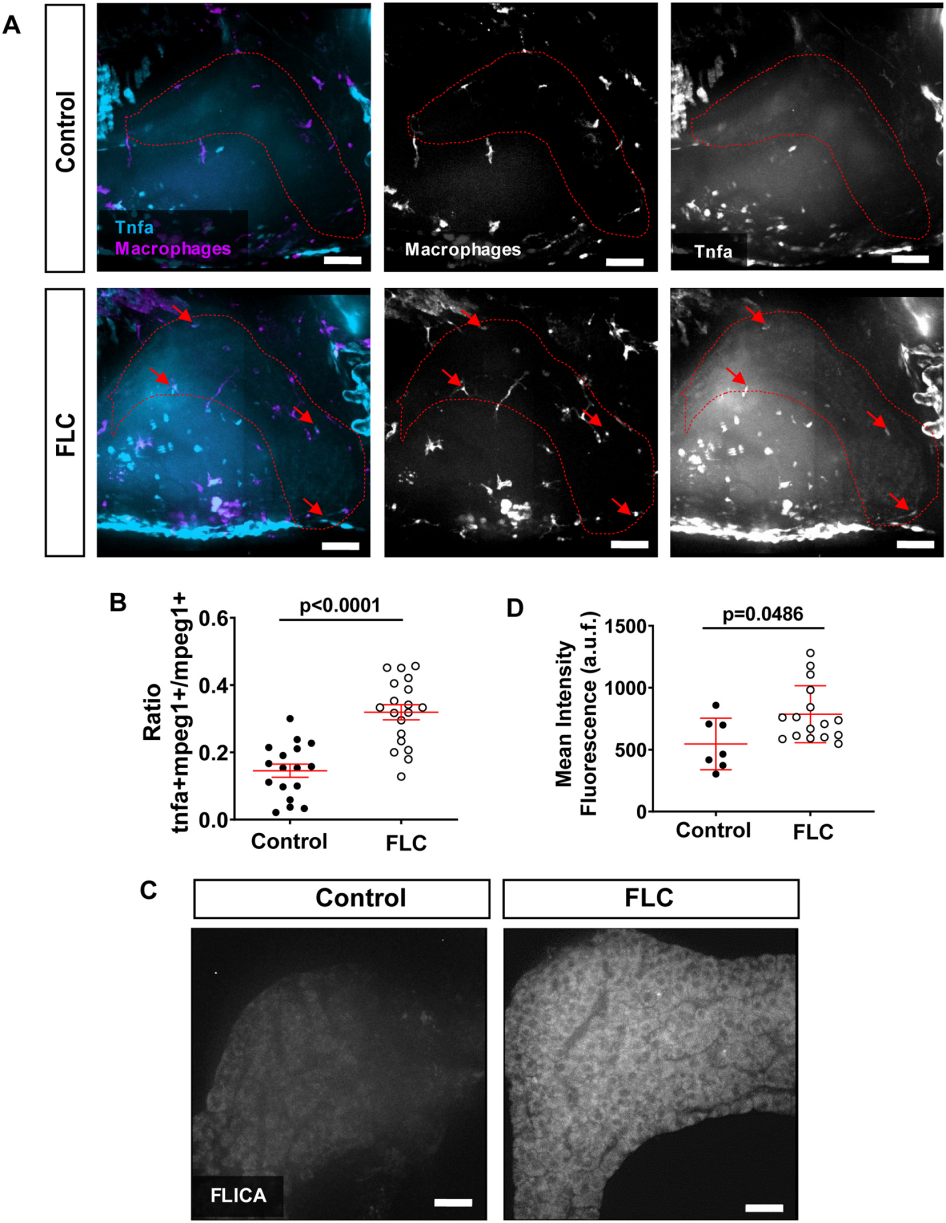
**FLC larvae show increased pro-inflammatory macrophages and increased caspase-a activity** **in** **the liver area.** (A) Representative MIPs of 7 dpf FLC larvae [*Tg(fabp10a:dnajb1a-prkacaa_cryaa;Cerulean)/Tg(tnfa:egfp)/Tg(mpeg1:mCherry-caax)*] and control siblings [*Tg(tnfa:egfp)/Tg(mpeg1:mCherry-caax)*]. Dotted red lines indicate liver area; red arrows indicate Tnfα-positive macrophages. (B) Graph showing ratio of Tnfα-positive macrophages to total macrophages in the liver area (control *N*=17, FLC *N*=19). (C) Representative MIPs of 7 dpf FLC larvae [*Tg(fabp10a:dnajb1a-prkacaa_cryaa;Cerulean)*] and control wild-type siblings. (D) Graph showing mean intensity fluorescent quantification in the liver area (control *N*=7, FLC *N*=16). Scale bars: 20 μm. Data are at least two independent experiments. Analysis performed in EMM in R. Dot plots show mean±s.e.m.; *P*-values are shown on graphs. a.u.f., arbitrary units of fluorescence.

In our previous work, we found that the presence of pro-inflammatory macrophages in the liver microenvironment at early stages of progression in hepatocellular carcinoma (HCC) larvae is associated with increased tumorigenesis in a model of non-alcoholic fatty liver disease (NAFLD)-associated HCC (de Oliveira et al., 2019). Nothing is known about the effect of DnaJ-PKAc on macrophage polarization *in vivo*. To identify pro-inflammatory macrophages, we outcrossed the FLC transgenic line, *Tg(fabp10a:dnajb1a-prkacaa_cryaa:Cerullean)*, with a reporter line of Tnfα expression, *Tg(tnfa:egfp)* (Table 1)*.* Tnfα is a key molecular player in liver disease progression (Jang et al., 2014) and is mostly expressed by resident macrophages in the liver (Nakashima et al., 2013; Tosello-Trampont et al., 2012). We found that FLC transgenic larvae have increased numbers of TNFα-positive macrophages compared to control siblings (Fig. 5A,B). In a recent study with a FLC murine model, a single-sample gene set enrichment analysis for select functionally annotated gene sets showed an upregulation of genes associated with the inflammasome complex (Kastenhuber et al., 2017). We therefore sought to determine if zfDnaJa-Pkaca induces inflammasome activation via the activation of Caspase-a, the zebrafish homolog for human caspase-1 (Angosto et al., 2012; Tyrkalska et al., 2016). Using FAM-FLICA assay, we found that Caspase-a activity is significantly increased in the liver of FLC transgenic larvae compared to controls (Fig. 5C,D). Overall, our data suggest that zfDnaJa-Pkaca promotes a pro-inflammatory liver microenvironment at early stages of FLC progression.

### Pharmacological inhibition of TNFα secretion and caspase-a activity decreases inflammation and FLC progression

**Fig. 6.**
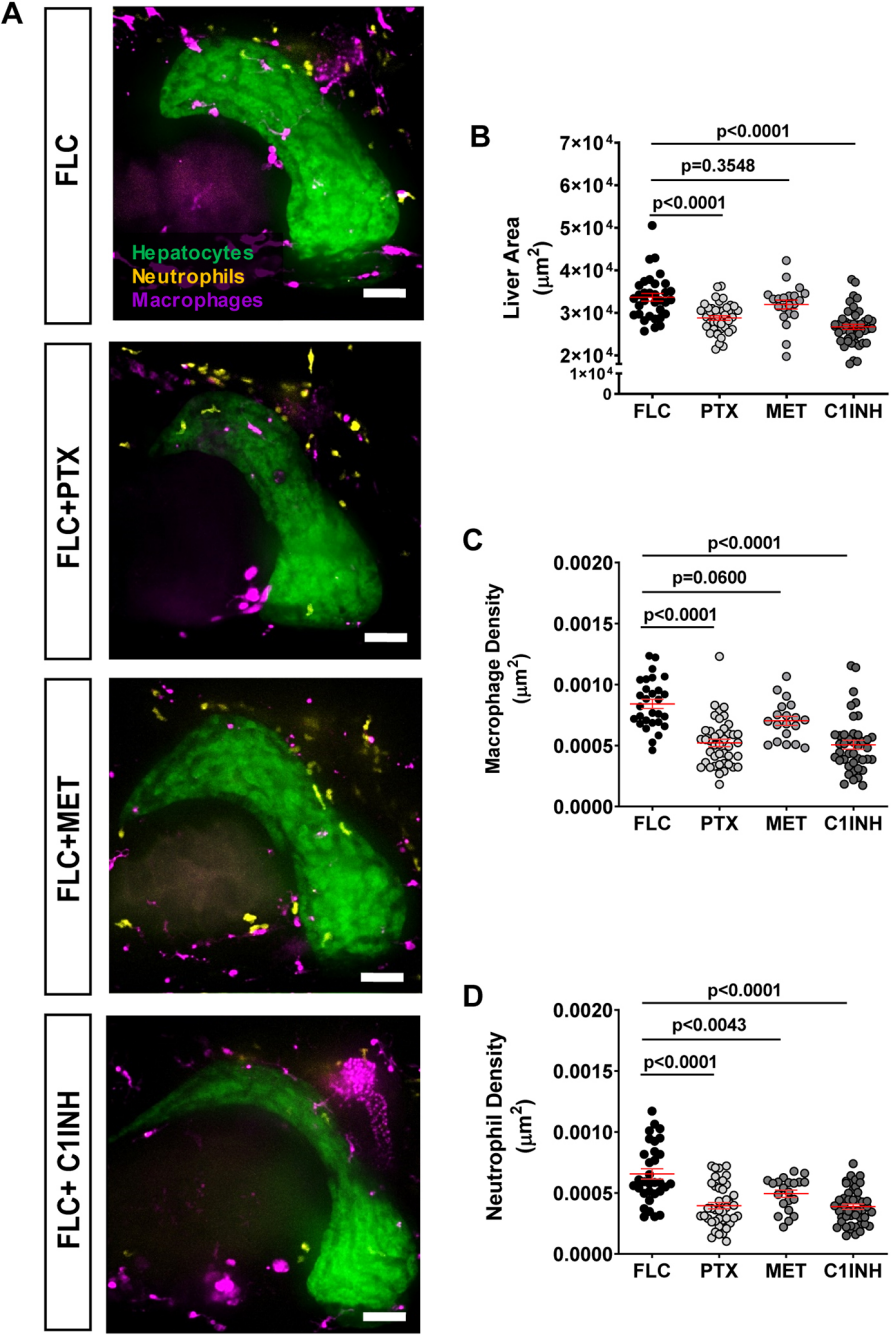
**Pharmacological inhibition of TNFα secretion and Caspase-a activity reduces inflammation and FLC progression.** (A) Representative MIPs of 7 dpf FLC larvae [*Tg(fabp10a:dnajb1a-prkacaa)/Tg(fabp10a:egfp-l10a)/Tg(lyzc:bfp)/Tg(mpeg1:mCherry-caax)*] and control siblings [*Tg(fabp10a:egfp-l10a)/Tg(lyzc:bfp)/Tg(mpeg1:mCherry-caax)*] treated with 50 μM pentoxifylline (PTX), 50 μM metformin (MET) and 100 μM Ac-YVAD-CMK (C1INH). (B-D) Graphs showing liver area (B) (control *N*=32, PTX *N*=42, MET *N*=21, C1NH *N*=46), macrophage density (C) (control *N*=29, PTX *N*=42, MET *N*=19, C1NH *N*=42) and neutrophil density (D) (control *N*=32, PTX *N*=42, MET *N*=21, C1NH *N*=46). Scale bars: 40 μm. Data are from at least three independent experiments. Analysis performed in EMM in R. Dot plots show mean±s.e.m.; *P*-values are shown on graphs.

Zebrafish larvae provide a powerful tool for drug screening (Wiley et al., 2017). Therefore, we used a pharmacological approach to identify small molecules that affect inflammation and early progression in our zebrafish model of early FLC. We found an increase in pro-inflammatory macrophages (Tnfα positive) and Caspase-a activity in the liver of FLC larvae (Fig. 4). Therefore, we next tested if inhibition of Tnfα secretion and Caspase-a with pentoxifylline (PTX) and Ac-YVAD-CMK (C1INH), respectively, affected innate immune cell infiltration and liver size in FLC transgenic larvae. We observed that both treatments significantly decreased liver size as well as macrophage and neutrophil recruitment to the liver of FLC transgenic larvae (Fig. 6A-D). We also tested the effects of metformin on FLC transgenic larvae. We and others have shown that metformin decreases inflammation and associated liver disease progression (de Oliveira et al., 2019; Li et al., 2019; Satapati et al., 2015). Surprisingly, we found that metformin treatment of FLC larvae did not affect liver size or macrophage infiltration (Fig. 6A-C). However, a small decrease in neutrophil infiltration was observed in FLC larvae treated with metformin (Fig. 6A,D). Overall, our data suggest that Tnfα and Caspase-a mediate FLC-associated liver inflammation and may represent new targets to limit FLC progression.

## DISCUSSION

FLC is a rare pediatric liver cancer with few effective therapeutic options. The fusion protein DnaJ-PKAc has been identified as a unique driver of FLC (Graham et al., 2015; Honeyman et al., 2014; Simon et al., 2015). Here, we report a zebrafish model for FLC generated by hepatocyte-specific ectopic expression of the zebrafish form of DnaJ-PKAc, zfDnaJa-Pkaca. We find that overexpression of zfDnaJa-Pkaca induces early hepatomegaly and inflammation in the liver area. One striking feature is that onset of inflammation occurs within the first few days post-fertilization and is characterized by the presence of Tnfα-positive macrophages, a feature that is not present in the standard catenin model of HCC (de Oliveira et al., 2019). In addition, this model provides a powerful tool to identify small molecules that alter inflammation and liver enlargement induced by zfDnaJa-Pkaca.

Our findings show that FLC transgenic fish develop masses, suggesting that, as in murine models, overexpression of *DnaJa-Pkaca* is sufficient to drive tumorigenesis *in vivo*. However, the incidence of mass formation was surprisingly low (1/8 at 8 months of age and 1/7 at 12 months). This may be due to low expression level or use of only one homolog of the *DNAJB1* and *PRKACA* genes. Although zfDnaJa-Pkaca has high identity and similarity with the human counterpart DnaJ-PKAc, it might not be enough to fully recapitulate the human disease in zebrafish. The beta form, zfDnajb-Pkacb, may also have an important role in the zebrafish liver, acting in parallel with the alpha form. It would be interesting to investigate whether the incidence of FLC tumorigenesis in zebrafish is increased by overexpression of both the alpha and beta forms of zfDnaJ-Pkac. It is also possible that the hepatocyte promoter *fabp10a* may not be sufficient to drive disease. Transcriptomic analysis of FLC human tissue has revealed a gene signature that closely resembles that of biliary tree stem cells (Oikawa et al., 2015), identified as hepatocyte precursors (De Assuncao et al., 2017; Español-Suñer et al., 2012; He et al., 2014). Future studies should use drivers in the biliary tree stem cells/liver progenitor cells to express the fusion transcript and test their effects on oncogenic potential. In addition, as in the murine model, the masses found in FLC transgenic zebrafish lack markers of fibrosis seen in human FLC. This feature might be a crucial step for the progression of the disease and might be achieved with the use of fibrotic stimulants (Kastenhuber et al., 2017). Activation of additional signaling pathways such as WNT/β-catenin (Kastenhuber et al., 2017) might also be used to enhance zfDnaJ-Pkac tumorigenesis *in vivo* in the zebrafish model.

The function and activity of DnaJ-PKAc has been another major focus of study in the field, since drugs that target PKA activity could be a potential therapeutic option for FLC patients. Up to now, most data suggest that DnaJ-PKAc fusion is enzymatically active and necessary for tumorigenesis *in vivo* (Kastenhuber et al., 2017). PKA activity mostly exerts an anti-inflammatory role (Campo et al., 2012) and is a major regulator of innate immune cells (Serezani et al., 2008). Several clinical drugs that target the cAMP/PKA signaling pathway increase cAMP and are used to reduce inflammation and treat inflammatory disorders (Banner and Trevethick, 2004; Serezani et al., 2008). Importantly, immune cells are a main source of trophic support for transformed cancer cells and can play a role in the early progression of cancer (Feng et al., 2010; Giese et al., 2019; Powell and Huttenlocher, 2016), including liver cancer (Yan et al., 2015, 2017; Zhao et al., 2016). The role of DnaJ-PKAc in the modulation of the immune cell composition of the liver microenvironment is understudied. Using fluorescent-labeled transgenic zebrafish larvae as a model, we and others are able to visualize the early interactions between immune cells and transformed hepatocytes to study the immune mechanisms involved in liver disease and cancer progression through non-invasive live imaging (de Oliveira et al., 2019; Yan et al., 2015; Yan et al., 2017). As discussed above, the prediction would be that the zfDnaJa-Pkaca would have an inhibitory impact on the innate immune response. Surprisingly, we observed the opposite – increased innate immune inflammation in our FLC transgenic model, with increased neutrophil and macrophage infiltration into the liver, as well as an increase in Tnfα-positive macrophages. These data suggest that zfDnaJa-Pkaca promotes a pro-inflammatory liver microenvironment. Indeed, such an effect might not be surprising in the context of FLC. The presence of uncontrolled fibrosis (Ward and Waxman, 2011), NF-κB activation (Li et al., 2009) and increased levels of CD68 (Ross et al., 2011), a cytoplasmic marker of macrophage and neutrophil granules (Ross et al., 2011), suggest the presence of leukocytes in the liver microenvironment of FLC patients. Moreover, the heterogeneous activation of the ERK signaling cascade promoted by the association of Hsp70 with DnaJ-PKAc (Turnham et al., 2019) may also promote the upregulation of pro-inflammatory genes through activation of the NF-κB transcription factor or other mechanisms (Li et al., 2009). Another pro-inflammatory pathway that might induce the pro-inflammatory microenvironment found in the liver in FLC larvae is the dual oxidase 1 (DUOX1)/hydrogen peroxide (H_2_O_2_)/NF-κB pathway (Candel et al., 2014; de Oliveira et al., 2015, 2014). DUOX1 is positively regulated by the cAMP/PKA cascade (Rigutto et al., 2009). This NADPH oxidase is a main source of reactive oxygen species (ROS) production, such as H_2_O_2_, in epithelial tissues, including the liver. DUOX1 is overexpressed in liver tumors and further has been identified as a potential prognostic marker in HCC patients (Chen et al., 2016; Lu et al., 2011). H_2_O_2_ induces leukocyte recruitment and an inflammatory response after tissue damage (Candel et al., 2014; de Oliveira et al., 2015, 2014; Niethammer et al., 2009; Razzell et al., 2013; Yoo et al., 2011), as well as at early stages of cancer progression (Feng et al., 2010). Interestingly, murine and human FLC tumors show upregulation of genes associated with ROS pathways, including enzymes involved in detoxifying ROS (Kastenhuber et al., 2017). It would be interesting to investigate whether DUOX1/H_2_O_2_ signaling pathways are involved in the observed pro-inflammatory effect of zfDnaJa-Pkaca in our FLC zebrafish model. Moreover, we found increased Caspase-a activity in the liver of FLC larvae, indicating increased activation of the inflammasome, which is in agreement with previous findings in human and murine FLC tumors (Kastenhuber et al., 2017).

Recurrence after complete surgical resection is common in FLC patients (Kassahun, 2016). The therapeutic strategies available for these patients with FLC relapse are limited and often not effective. Therefore, the discovery of new and improved therapeutic targets is a primary goal in the field. FLC zebrafish models could be powerful tools to aid in the identification of new drug targets using small-molecule screening. Importantly, zebrafish models display similar features of liver cancer progression to those of humans (Goessling and Sadler, 2015; Huo et al., 2019; Lam et al., 2006; Li et al., 2014, 2013; Wrighton et al., 2019; Zheng et al., 2013). We found that inhibition of Tnfα secretion and Caspase-a activity both reduced innate immune cell infiltration in the liver as well as liver size, an established marker for liver disease progression (de Oliveira et al., 2019; Evason et al., 2015; Lam et al., 2006; Nguyen et al., 2012; Zheng et al., 2014). In addition to PTX and C1INH, we tested the effects of metformin on FLC. Metformin directly inhibits mitochondria complex I, increasing hepatic AMPK, and can also indirectly decrease hepatic cAMP levels and consequently dampen PKA activity (Pernicova and Korbonits, 2014). Although metformin controls liver inflammation in a zebrafish model of NAFLD associated with HCC (de Oliveira et al., 2019), metformin did not significantly affect liver size or inflammation in larval FLC. These findings support the idea that targeting PKA activity alone is not enough to suppress the oncogenic effect of DnaJ-PKAc. It is likely that an important part of DnaJ-PKAc oncogenic effect is through the scaffolding function and impact on ERK signaling via DnaJ-PKAc/Hsp70 macromolecular assembly (Turnham et al., 2019). Combination therapies targeting PKA activity, ERK signaling and inflammation in FLC may represent an attractive approach. Future studies using combination treatments can easily be tested in the zebrafish model of FLC.

Here, we report a new FLC zebrafish model with unmatched non-invasive live-imaging capabilities and scalability amenable to high-throughput drug screening. Overall, our findings support the idea that non-resolving inflammation might be fueling the liver microenvironment and contributing to FLC pathology. In addition, we found that pharmacological inhibition of TNFα secretion and caspase-1 activity might be targets to treat inflammation and progression in FLC patients. In the future, it will be interesting to address how the FLC pro-inflammatory liver environment modulates the adaptive immune system; zebrafish models might be key tools in unraveling such mechanisms and finding new and improved therapeutic targets for FLC.

## MATERIALS AND METHODS

### Zebrafish husbandry and maintenance

All protocols using zebrafish in this study were approved by the University of Wisconsin-Madison Institutional Animal Care and Use Committee. Adult zebrafish and embryos up to 5 dpf were maintained as described previously (de Oliveira et al., 2019). At 5 dpf, larvae were transferred to 15-cm Petri dishes and kept in E3 medium without Methylene Blue until 7 dpf. For all experiments, larvae were anesthetized in E3 medium without Methylene Blue containing 0.16 mg/ml tricaine (MS222/ethyl 3-aminobenzoate; Sigma-Aldrich). Zebrafish lines used are summarized in Table 1.

### Generation of *Tg(fabp10a:dnajb1a-prkacaa_cryaa:Cerulean)*, *Tg(fabp10a:egfp-l10a)* and *Tg(fabp10a:mcherry-kras)* lines

For *Tg(fabp10a:dnajb1a-prkacaa_cryaa:Cerulean)*, DNA coding sequence for the *dnajb1a-prkacaa* fusion gene was PCR amplified from a plasmid synthetized by Integrated DNA Technologies using the following primers: Forward, 5′-CTTTGTGTTGATCGGGTACCGCCACCATGGGAAAAGATT-3′; Reverse, 5′-CTGATTATGATCTAGACTAGAATTCAGCAAACTCCT-3′.

The resulting PCR products were gel purified, and cloned using an InFusion kit (Clontech) into an expression vector containing the *fabp10a* promoter, minimal Tol2 elements for efficient integration and an SV40 polyadenylation sequence (Yoo et al., 2012), previously digested with KpnI and XbaI and gel purified. F0 Casper larvae were obtained by injecting 3 nl of 12.5 ng/ml DNA plasmid and 17.5 ng/ml *in vitro* transcribed (Ambion) transposase mRNA into the cell of a one-cell-stage embryo. F0 larvae were raised to breeding age and crossed to adult Casper zebrafish. Founders were screened for Cerullean-positive eye using a Zeiss Axio Zoom stereomicroscope (EMS3/SyCoP3; Zeiss; PlanNeoFluar Z 1×:0.25 FWD 56 mm lens).

For *Tg(fabp10a:egfp-l10a)* DNA coding, *egfp-l10a* was PCR amplified from a plasmid (Davenport et al., 2016), using the following primers: Forward, 5′-CTTTGTGTTGATCGggtaccGCCACCATGGTGAGCAAGGGCGAGGA-3′; Reverse, 5′-CTGATTATGATCTAGACTAATACAGACGCTGGGGCTTGC-3′.

The resulting PCR products were gel purified and cloned using an InFusion kit (Clontech) into an expression vector containing the *fabp10a* promoter sequence, minimal Tol2 elements for efficient integration and an SV40 polyadenylation sequence, previously digested with KpnI and XbaI and gel purified. Casper fish were injected and screened for EGFP expression in the liver as described above.

For *Tg(fabp10a:mCherry-kras)* DNA coding, *kras* was PCR amplified from a plasmid (Freisinger and Huttenlocher, 2014) using the following primers: Forward, 5′-ACGAGCTGTACAAGTCCGGAATGACTGAATATAAACTTGTGGTGGTG-3′; Reverse, 5′-CTGATTATGATCTAGATTACATAATTACACACTTTGTCTTTGACTTCT-3′.

The resulting PCR products were gel purified and cloned using an InFusion kit (Clontech) into an expression vector containing the *fabp10a* promoter sequence, mCherry sequence, minimal Tol2 elements for efficient integration and an SV40 polyadenylation sequence, previously digested with BspEI and XbaI and gel purified. Casper fish were injected and screened for mCherry expression in the liver as described above.

### Liver dissection and histology

Adult zebrafish (8 and 12 months of age) were euthanized by tricaine overdose and the livers removed by dissection. Livers were fixed in 10% formalin overnight. Samples were coded to facilitate blinded histopathologic evaluation. The livers were paraffin embedded and 4-μm sections were prepared and stained with H&E. Slides were evaluated by a board-certified veterinary pathologist (R.A.H.).

### Live imaging

All live imaging was performed using a zWEDGI device as previously described (Huemer et al., 2017). For time-lapse imaging, the loading chamber was filled with 1% low-melting-point agarose (Sigma-Aldrich) in E3 medium to retain the larvae in the proper position. Additional tricaine/E3 medium was added as needed. All images were acquired with live larvae with the exception of FLICA staining. Images were acquired on a spinning disk confocal microscope (CSU-X; Yokogawa) with a confocal scanhead on a Zeiss Observer Z.1 inverted microscope equipped with a Photometrics Evolve EMCCD camera using a Plan-Apochromat 20×/0.8 NA M27 air objective with a 5-μm interval. For larvae with large livers, 2×2 tile images were taken.

### Liver and hepatocyte size measurements

For liver size measurements, 7 dpf *Tg(fabp10a:dnajb1a-prkacaa_cryaa:Cerulean)* larvae were outcrossed with *Tg(fabp10a:egfp-l10a)* larvae. For hepatocyte measurements, *Tg(fabp10a:dnajb1a-prkacaa_cryaa:Cerulean)* larvae were outcrossed with *Tg(fabp10a:mCherry-kras)* larvae*.* Liver area, liver volume, hepatocyte area and hepatocyte diameter were measured as previously described (de Oliveira et al., 2019). For hepatocyte measurements, 20 cells per larva were analyzed.

### Quantification of neutrophil and macrophage recruitment

To quantify leukocyte recruitment, we outcrossed the double-transgenic FLC line carrying EGFP-L10a as a liver marker, *Tg(fabp10a:dnajb1a-prkacaa_cryaa:Cerulean)*/*(fabp10a:egfp-l10a)*, with a double-transgenic line with labeled macrophages and neutrophils, *Tg(mpeg1-mCherry-caax/lyzc:bfp)*. After live imaging, Z-series images were reconstructed in 2D maximum-intensity projections (MIPs) on ZEN pro 2012 software (Zeiss). Neutrophils and macrophages were counted within 50 μm from the liver. The area of the liver was measured in each larva and used to normalize the number of innate immune cells per liver area.

### TNFα-positive macrophages imaging and quantification

To assess TNFα-positive macrophages in the liver area, we outcrossed the FLC line, *Tg(fabp10a:dnajb1a-prkacaa_cryaa:Cerulean)*, with a double-transgenic line labeled for macrophages and expressing EGFP under the TNFα promoter, *Tg(mpeg1:mCherry-caax/tnfa:egfp)* (Miskolci et al., 2019). After live imaging, Z-series images were 3D reconstructed on Imaris software. Using the Imaris spots tool, total macrophages (*mpeg1:mCherry-caax*-positive cells) were counted within 50 μm from liver. TNFα-positive macrophages (double-positive *mpeg1:mcherry/tnfa:egfp* cells) were quantified similarly. Z-series images were reconstructed on Zen software to create MIPs.

### Drug treatment

Larvae were treated with metformin, PTX and C1INH as described previously (de Oliveira et al., 2019; Tyrkalska et al., 2016). Briefly, we dissolved metformin (Enzo Life Sciences) in E3 medium without Methylene Blue at a final concentration of 50 µM. PTX and C1INH were first reconstituted in dimethyl sulfoxide and later diluted 1000× in E3 medium without Methylene Blue at a final concentration of 50 µM and 100 µM, respectively. Larvae were treated with these drugs from 3 dpf to 7 dpf. Drugs were freshly prepared and replaced daily.

### Caspase-a activity assay

Larvae at 6 dpf were incubated with FAM-FLICA (Immunochemistry Technologies) at 1:300 for 12 h. The next day, larvae were washed with E3 medium and fixed in PIPES/1.5% formaldehyde buffer at 4°C overnight. Larvae were washed three times with PBS 1×, and livers were carefully dissected with the use of forceps on a stereomicroscope (Leica MZ 9.5). After dissection, livers were immersed in PBS and images were acquired on a spinning disk confocal microscope (CSU-X; Yokogawa) with a confocal scanhead on a Zeiss Observer Z.1 inverted microscope equipped with a Photometrics Evolve EMCCD camera using an EC Plan-Neofluor 40×/0.75 NA M27 air objective with a 1-μm interval. Mean fluorescence intensity was measured from MIPs using Image J.

### Statistical analysis

All data plotted comprise at least three independent experimental replicates. Estimated Marginal Means (EMM) analysis in R (www.r-project.org) (Vincent et al., 2016) was performed on pooled replicate experiments, using Tukey method when comparing more than two treatments. Graphical representations were done in GraphPad Prism version 6.

## Supplementary Material

Supplementary information
