# Supplementary material for: DnaJ-PKAc fusion induces liver inflammation in a zebrafish model of fibrolamellar carcinoma
Source: Dis Model Mech. 2020 Apr 30;13(4):dmm042564. doi: 10.1242/dmm.042564 (PMC7197716; doi:10.1242/dmm.042564)
Supplement: Supplementary information [file dmm-13-042564-s1.pdf]

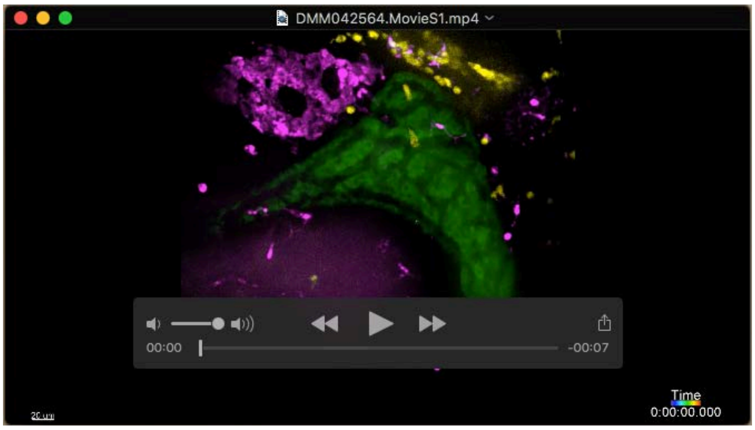

Movie 1: Time-lapse movies of leukocyte recruitment to liver area of 7-day post fertilization control larvae. Macrophage (magenta); neutrophil (yellow); Hepatocytes (green).

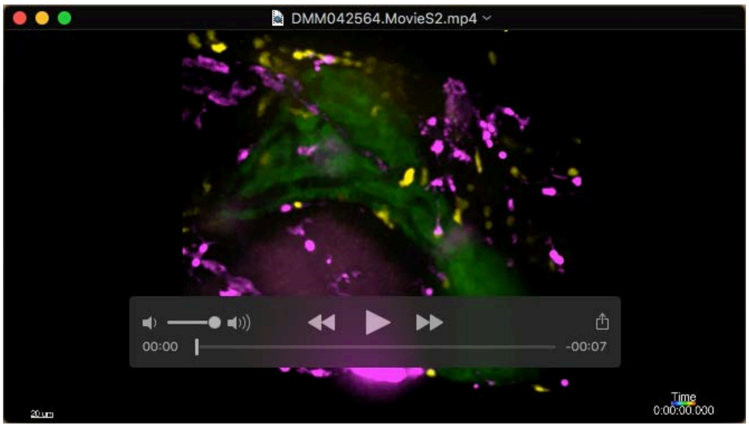

Movie 2: Time-lapse movies of leukocyte recruitment to liver area of 7-day post fertilization transgenic FLC larvae. Macrophage (magenta); neutrophil (yellow); Hepatocytes (green).

Disease Models & Mechanisms • Supplementary information
